# Response of soil microbial community structure to temperature and nitrogen fertilizer in three different provenances of *Pennisetum alopecuroides*

**DOI:** 10.3389/fmicb.2024.1483150

**Published:** 2024-10-23

**Authors:** Niandong Deng, Lili Nian, Shuolun Zhang, Yixuan Liang, Huiying Shang, Yang Li, Zhuxin Mao

**Affiliations:** ^1^College of Geology and Environment, Xi’an University of Science and Technology, Xi’an, China; ^2^Gansu Academy Agricultural Sciences, Lanzhou, China; ^3^Xi’an Botanical Garden of Shaanxi Province, Institute of Botany of Shaanxi Province, Xi’an, China; ^4^Shaanxi Engineering Research Centre for Conservation and Utilization of Botanical Resources, Xi’an, China

**Keywords:** *Pennisetum*, soil microbes, ecological networks, nitrogen addition, temperature

## Abstract

Soil microorganisms are key indicators of soil health, and it is crucial to investigate the structure and interactions of soil microbial communities among three different provenances of *Pennisetum alopecuroides* under varying nitrogen fertilizer and temperature levels in Northwest China. This study aims to provide theoretical support for the sustainable use of artificial grassland in this region. Employing a two-factor pot-control experiment with three nitrogen fertilizer treatments and three temperature treatments, a total of all treatments was utilized to examine the composition and abundance of soil microbial communities associated with *Pennisetum alopecuroides* using high-throughput sequencing, PCR technology, and molecular ecological network analysis. The results revealed that *Proteobacteria* was the dominant bacterial phylum while *Ascomycota* was the dominant fungal phylum in the soil samples from three provenances of *Pennisetum*. Specifically, *Proteobacteria* exhibited higher abundance in the N3T2 treatment compared to other treatments under N3T2 (25–30°C, 3 g/pot) treatment conditions in Shaanxi and Gansu provinces; similarly, *Proteobacteria* was more abundant in the N1T2 (25–30°C, 1 g/pot) treatment in Inner Mongolia under N1T2. Moreover, Ascomycota displayed higher abundance than other treatments in both Inner Mongolia and Gansu provinces. Additionally, *Pennisetum Ascomycota* demonstrated greater prevalence under (25–30°C, 3 g/pot) treatment compared to other treatments; furthermore, Shaanxi’s *Pennisetum Ascomycota* exhibited increased prevalence under N3T1 (18–23°C, 3 g/pot) treatment compared to other treatments. The richness and diversity of soil microbial communities were significantly influenced by nitrogen fertilizer and temperature changes, leading to notable alterations in their structure. Molecular ecological network analyses revealed strong collaborative relationships among microbial species in Shaanxi *Pennisetum* and Inner Mongolia *Pennisetum* under high nitrogen and high temperature treatments, while competitive relationships were observed among microbial species in Gansu *Pennisetum* under similar conditions. Redundancy analysis indicated that soil pH, total potassium, and total phosphorus were the primary environmental factors influencing microorganisms. In summary, this study offers a theoretical foundation for assessing the sustainable utilization of *Pennisetum* artificial grasslands in Northwest China by investigating the shifts in soil microbial communities and the driving factors under varying nitrogen fertilizer and temperature levels.

## 1 Introduction

Soil microbial communities have received considerable attention as valuable indicators of soil biodiversity and environmental quality (Bertola et al., 2021). Soil microorganisms exhibit a remarkable diversity and abundance of species, occupying a pivotal role within soil ecosystems (Yan et al., 2020; Yao and Zhou, 2022). Highly responsive to environmental changes, soil microbial communities undergo shifts in composition and diversity in response to changes in aboveground plant communities and soil environment, as well as alterations in the soil ecological environment (Liu et al., 2023; Shen et al., 2024; Wen et al., 2024). This connection facilitates the material cycling and energy flow between aboveground and underground ecosystems (Graham and Knelman, 2023). Among soil microorganisms, bacteria constitute the most abundant and versatile group versatile group, playing a pivotal role in soil formation, organic matter decomposition, and nutrient cycling (Zhan et al., 2024). Fungi not only play a crucial role in apoplastic decomposition and nutrient cycling, but also contribute significantly to soil formation and nutrient cycling (Xie et al., 2024). Moreover, fungi establish symbiotic relationships with higher plants, thereby providing essential nutrients for plant growth (Wang et al., 2022). The study revealed a significant increase in soil temperature due to warming (Seaton et al., 2022). Additionally, it was observed that warming significantly enhanced soil microbial biomass but resulted in a notable decrease in microbial diversity and alterations in community structure (Seaton et al., 2022). Conversely, no impact of warming on soil microbial community composition was detected (Zhang et al., 2020). Another aspect of the study demonstrated that warming induced elevated evaporation of soil water, leading to reduced soil moisture and subsequently diminished effectiveness of soil nutrients. Consequently, this reduction negatively affected soil microbial biomass and activity (Goncharov et al., 2023; Shao et al., 2023). Studies consistently indicate that nitrogen addition generally diminishes microbial diversity (Wang et al., 2018), with a decreasing trend observed as nitrogen input increases (Liu et al., 2020). Molecular ecological network analysis is a widely employed approach for characterizing soil microbial symbiosis patterns by elucidating network structural features and unveiling interactions among diverse soil microbial taxa (Layeghifard et al., 2017). Generally, increased connectivity and complexity of the network indicate active involvement of soil microbes in the ecosystem (Jiao et al., 2022). Moreover, the complexity of soil microbial networks is closely linked to their stability. Soil microorganisms maintain relative stability of the network to withstand environmental disturbances. Negative correlations, which signify competition, often enhance network stability through negative feedback mechanisms (Duchenne et al., 2022). Therefore, molecular ecological network analysis can effectively unveil interrelationships’ complexity and stability among soil microbes while reflecting ecosystem intricacy and stability (Landi et al., 2018). It should be noted that soil microbial interactions are not static but exhibit significant spatial and temporal variability as well as sensitivity to environmental factors. Climate, altitude, and vegetation exert substantial influences on soil microbial interactions leading to variations in molecular ecological networks’ complexity and stability (de Vries et al., 2018).

Northwest China is a representative ecologically fragile region characterized by severe soil erosion, low vegetation cover, and prominent soil degradation issues (Li et al., 2024; Yu et al., 2024; Zhang et al., 2023). *Pennisetum alopecuroides*, a perennial herbaceous plant belonging to the Gramineae family, derives its name from its inflorescence resembling a wolf’s tail and has origins in western Australia and eastern Asia, including China (Kang et al., 2018; Wang et al., 2017). This species exhibits remarkable drought tolerance and adaptability while playing a crucial role in facilitating vegetation recovery, enhancing the ecological environment, and promoting local livestock development (Dang et al., 2017). However, suboptimal soils restrict the growth of *Pennisetums*, necessitating their adaptation to varying temperature zones due to significant altitudinal differences (Belesky and Malinowski, 2016). Consequently, this not only leads to abrupt declines in grass production and soil quality degradation but also exerts profound impacts on the structure and function of soil microbial communities. Thus, nitrogen fertilizers along with suitable temperature conditions are essential for sustaining its utilization (Yang et al., 2020). It has been suggested that moderate warming may enhance specific positive feedback mechanisms and promote network stability, whereas extreme temperatures could amplify negative correlations and result in network instability (Li et al., 2021). Addition of nitrogen fertilizer might improve network connectivity and stability; however, excessive nitrogen fertilizer application could intensify competitive relationships and disrupt network connectivity, leading to potential network instability (Widdig et al., 2020). The impact of temperature and N fertilizer addition on the stability of microbial communities in wolverine soils remains unclear. Therefore, investigating the dynamics of soil microbial community diversity patterns and network stability during temperature variations and N fertilizer addition in Northwest China will contribute to a comprehensive understanding of the formation and maintenance mechanisms underlying soil microbial community diversity patterns, as well as provide insights for guiding vegetation restoration efforts and biodiversity conservation.

Currently, numerous studies have been conducted in China on the characteristics of soil microbial communities of *Pennisetum* in Northwest China in response to temperature and nitrogen fertilizer addition. These studies primarily focus on soil microbial biomass, community composition and structure, exploring patterns of change with respect to temperature and nitrogen fertilizer, as well as their interrelationships with environmental factors. However, fewer studies have investigated co-occurrence patterns of soil microbial communities during temperature and nitrogen fertilizer addition for different seed sources of *Pennisetum* in Northwest China. Therefore, this study focuses on the composition and structural characteristics of soil microbial communities (fungi and bacteria) as well as changes in species co-occurrence patterns for three different seed sources of *Pennisetum* based on pot experiments. The aim is to provide a theoretical basis for sustainable use of artificial grasslands composed of *Pennisetum.*

## 2 Materials and methods

### 2.1 Test materials

**Figure 1:**
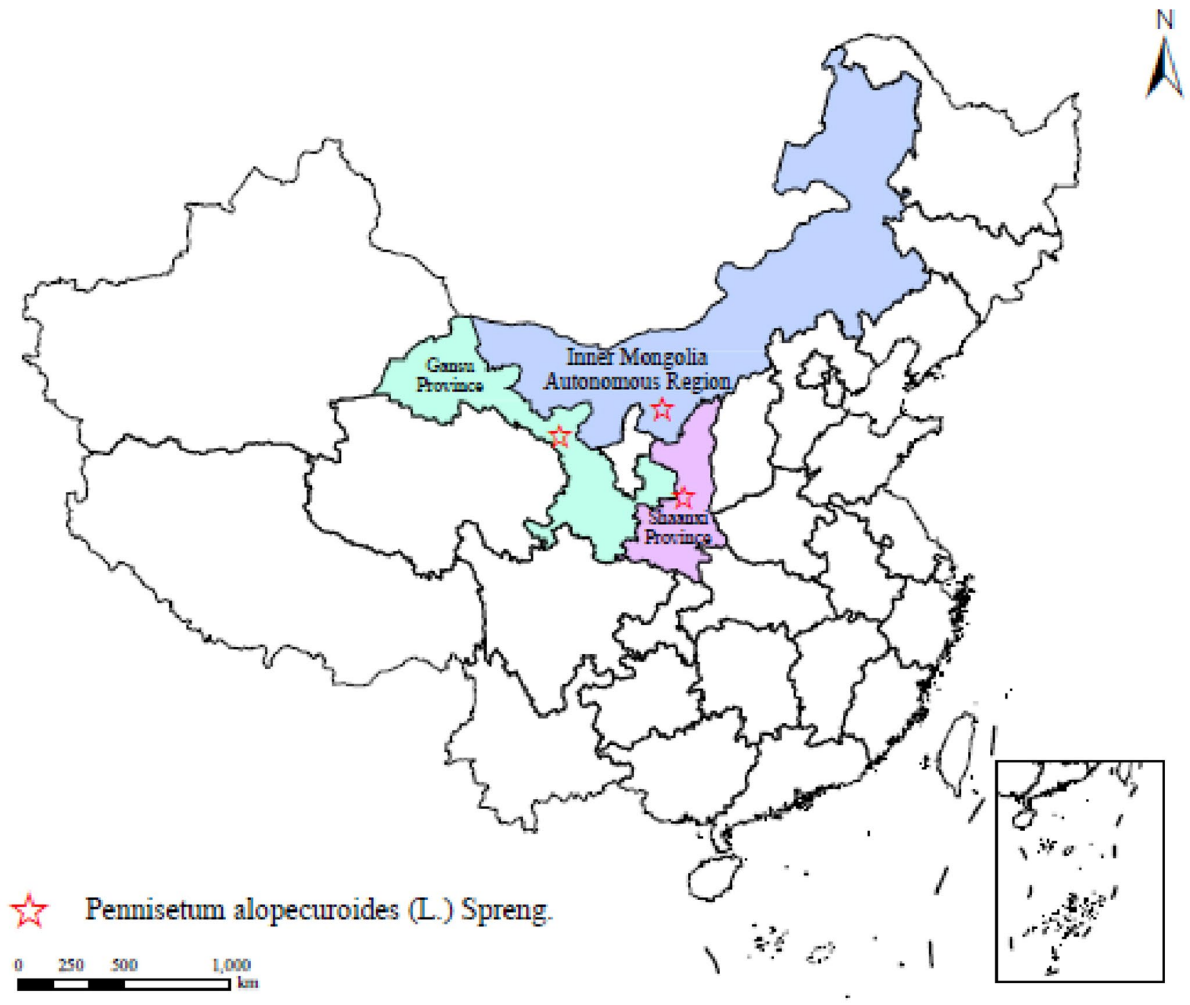
Distribution of experimental *Pennisetum alopecuroides* in China. This map shows the distribution of Pennisetum in China, marking three main planting areas: Gansu Province, Inner Mongolia Autonomous Region, and Shaanxi Province. Each area is marked with a different color on the map, with red stars indicating the distribution points of *Pennisetum* in the corresponding areas.

The test materials were *Poaceae* plants *Pennisetum alopecuroides*, and the seeds of the three provenances of *Pennisetum* sourced from Shaanxi Province, Inner Mongolia Autonomous Region and Gansu Province, respectively, (Figure 1). All seeds were mature and collected in 2023. We list key properties and characteristics of soil to provide context for subsequent microbial community analyses. Basic soil properties: pH 8.09, organic matter 15.62 g/kg, total nitrogen 0.75 g/kg, total phosphorus 0.76 g/kg, and total potassium 18.77 g/kg.

### 2.2 Experimental design

The experiment was a factorial design, consisting of three nitrogen fertilizer treatments and three temperature treatments. An orthogonal experimental design was employed, resulting in a total of nine treatments with three replicates for each treatment and 27 pots per plant (Table 1). Seedlings were raised in May 2023 and emerged uniformly after 15 days. Nitrogen fertilizer treatment commenced at the beginning of August, followed by greenhouse temperature treatment (Plants are grown in a greenhouse environment with consistent temperature and light conditions) at the end of August. The pots used were made from commercially available pp. material, measuring 265 mm in caliber, with a ground diameter of 155 mm and a height of 175 mm. The soil was a mix of field soil and river sand (3:1 volume ratio), thoroughly mixed and uniformly potted with 2,500 g per pot. Nitrogen was commercially available urea. N1 was 0.5 g/pot of urea (1 kg/ha of pure nitrogen^2^), N2 was 1 g/pot of urea (2 kg/ha of pure nitrogen^2^), and N3 was 1.5 g/pot of urea (3 kg/ha of pure nitrogen^2^), fully dissolved in water and added in batches. During the experimental period, fertilization was applied only once.

**Table 1:** Orthogonal experimental design.

Experiment No.	N1T1	N1T2	N1T3	N2T1	N2T2	N2T3	N3T1	N3T2	N3T3
Temperature (°C)	18–23	25–30	32–37	18–23	25–30	32–37	18–23	25–30	32–37
Urea (g/pot)	1	1	1	2	2	2	3	3	3

### 2.3 Soil sample collection

The seedling nursery work was conducted in the greenhouse. Once the seedlings had emerged uniformly, those with similar growth patterns were selected for transplantation. Temperature control and fertilization experiments were carried out using pots. Nitrogen fertilizer treatment commenced in early August, followed by relocation to the greenhouse at the end of August for further temperature control and fertilization treatments. After a 30-day period of temperature control and fertilization treatments, both plants and soil samples were collected. The plants were manually divided into aboveground and underground (root system) parts. The process involved the following steps: First, the plants were removed from the greenhouse, and the surrounding soil was gently cleared to expose the root system. The aboveground portion was then carefully separated from the root system using scissors. Both parts were subsequently washed separately to remove residual soil and impurities. Finally, the cleaned aboveground portion and root system were appropriately processed and frozen to preserve their physiological state for further analysis. Soil samples were divided into two parts: one part was filled into sterilized centrifuge tubes, placed in foam boxes equipped with ice packs, transported to the laboratory, and stored at −80°C for total soil DNA extraction; while the other part was brought back to the laboratory, placed in a cool and ventilated area to air dry naturally for subsequent determination of soil physicochemical properties.

### 2.4 Methods for determining soil physical and chemical indicators

Determination of Soil Organic Carbon Using the Potassium Dichromate External Heating Method (Nelson and Sommers, 1996) and total soil nitrogen using the semi-micro Kjeldahl method (Brookes et al., 1985). Total phosphorus was determined by the sodium hydroxide-molybdenum antimonide colourimetric method (Levine et al., 1954). Soil pH was determined by the water immersion potentiometric method (Guimaraes et al., 2020). The total soil potassium was determined by flame photometric method (Shen et al., 2019). Chlorophyll was determined by Seely’s chlorophyll assay method (Jordan and Vilter, 1991). The procedure was as follows: Plant leaves were first cut into pieces and placed in a mortar. An appropriate amount of 95% ethanol and a small amount of calcium carbonate were then added to thoroughly grind the leaves, allowing for chlorophyll extraction. The mixture was subsequently filtered, and the filtrate was collected. Chlorophyll in the filtrate produced maximum absorption peaks at 649 nm and 665 nm. The absorbance values at these two wavelengths were measured using a spectrophotometer, and the chlorophyll content was calculated based on a standard curve.

### 2.5 High-throughput sequencing of soil microorganisms

Genomic DNA extraction from soil samples: The genomic DNA was extracted using the Power Soil^®^ DNA kit, and the purity of the DNA was checked by running the gel through 1.0% agarose gel electrophoresis, and then stained with ethidium bromide and detected by a gel imaging system. PCR amplification: Bacterial 16S rRNA primers 515F (5′-GTGCCAGCMGCCGCGG-3′) and 907R (5’-CCGTCAATTCMTTTRAGTTT-3′) were used for amplification. 907R (5′-CCGTCAATTCMTTTRAGTTT-3′) (Yang et al., 2017) To carry out the V4-V5 region, fungal primers were used ITS1F (5′-CTTGGTCATTTAGAGGAAGTAA-3′) and ITS1R (5′-GCTGCGTTCTTCATCGATGC-3′) (Sinclair et al., 2015) PCR amplification of ITS region was carried out. PCR was performed using Trans Gen AP221-02: Trans StartFastpfu DNA Polymerase; PCR instrument: ABI Gene Amp^®^ Model 9,700; PCR products of the same sample were mixed and detected by 2% agarose gel electrophoresis using AxyPrep DNA Gel The PCR products were cut and recovered using AxyPrep DNA Gel Recovery Kit (AXYGEN), eluted with Tris HCl and detected by 2% agarose electrophoresis. The PCR products were detected and quantified by applying Quanti Fluor™-ST Blue Fluorescence Quantification System (Promega) with reference to the first quantification step of electrophoresis (Mueller et al., 2014) Illumina Miseq PE300 sequencing was used.

### 2.6 Data processing

Soil physicochemical properties and microbial community composition data were processed using SPSS26.0 and Exce12010, with significance analysis performed using one-way ANOVA and multiple comparisons (LSD method, *p* = 0.05). Soil microbial genus Spearman correlation coefficients R > 0.5 and significant *p* < 0.05 species OUT were selected for constructing protozoa community correlation networks using the “igraph” package in R software, with network visualization analysis conducted using Gephi0.9.2 software. Redundancy analysis (RDA) of microbial communities and soil environmental factors was performed using CANOCO5.0, with graphical modifications using Al (Adobe Illustrator CS6) software.

## 3 Results and analyses

### 3.1 Soil physico-chemical factors

**Figure 2:**
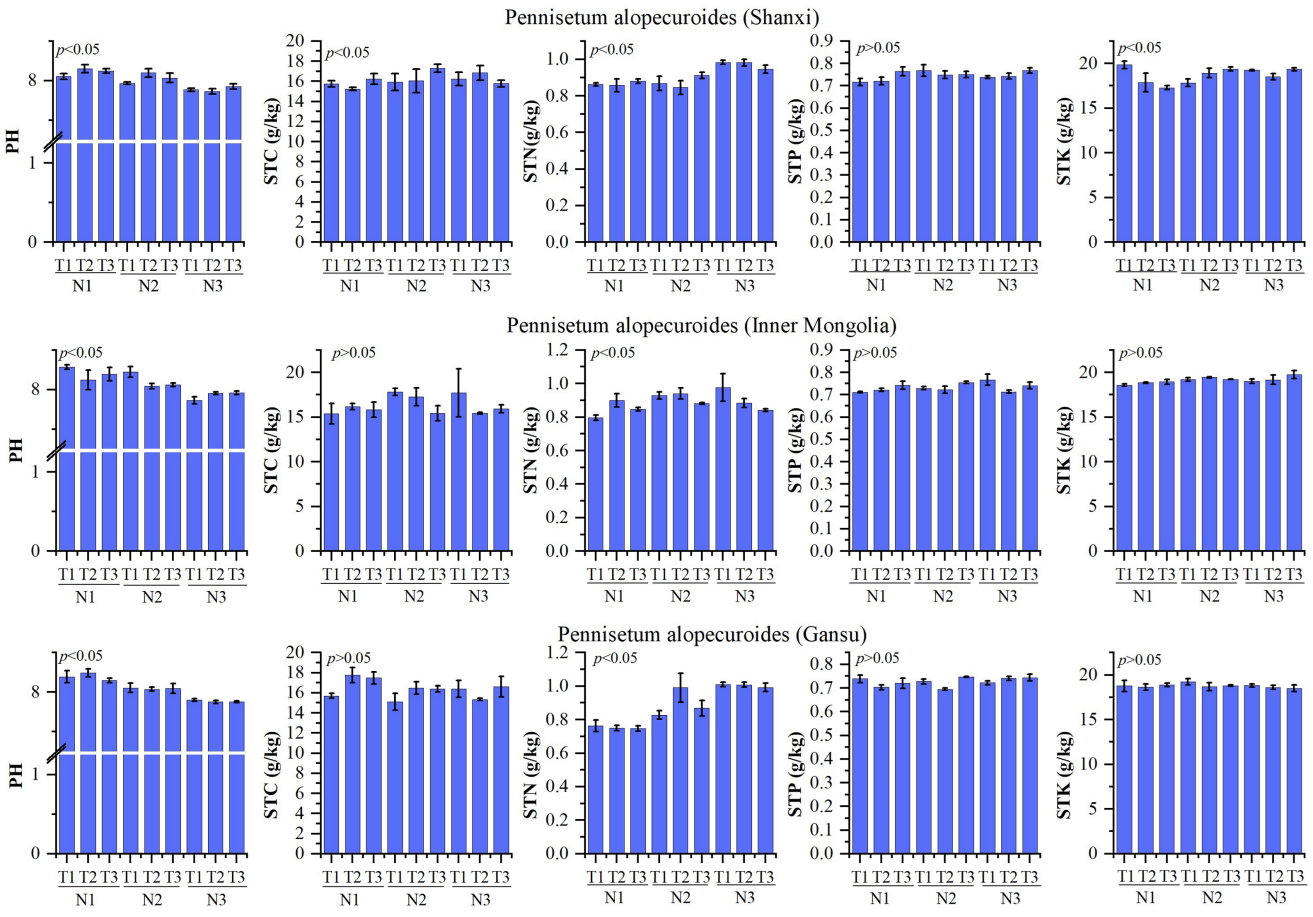
Physical and chemical properties of soil. N1-1 g/pot, N2-2 g/pot, N3-3 g/pot, T1-18/23°C, T2–25/30°C, T3: 32/37°C. *p* < 0.05: indicates that there is a significant difference between treatments, *p* > 0.05: indicates that there is no significant difference between treatments. The same below.

The physicochemical properties of the soil are presented in Figure 2. A study conducted on *Pennisetum* in Shaanxi revealed that changes in temperature and nitrogen fertilizer had no significant impact on soil total phosphorus, but significantly influenced pH, organic carbon, total nitrogen, and total potassium levels. The treatment N1T2 exhibited a significantly higher pH compared to other treatments (*p* < 0.05), while the N3T2 treatment showed a significantly lower pH than other treatments (*p* < 0.05). Organic matter content was significantly higher in the N2T3 treatment compared to other treatments (*p* < 0.05), whereas the N1T2 treatment displayed a significantly lower organic matter content than other treatments (*p* < 0.05). Total nitrogen levels were found to be significantly higher in the N3T2 treatment compared to other treatments (*p* < 0.05), with the N2T2 treatment exhibiting a significantly lower total nitrogen content than other treatments (*p* < 0.05). Additionally, the N1T1 treatment demonstrated a significantly higher total potassium level compared to other treatments, while the N1T3 treatment displayed a significantly lower total potassium level than other treatments (*p* < 0.05). Inner Mongolia *Pennisetum* study found that temperature and nitrogen changes had no significant effect on organic carbon, total phosphorus, and total potassium but significantly affected pH and total nitrogen. The pH of the N1T1 treatment exhibited a significantly higher value compared to other treatments, while the N3T1 treatment showed a significantly lower value (*p* < 0.05). Additionally, the total nitrogen content in the N3T1 treatment was significantly higher than that in other treatments, whereas the N1T1 treatment displayed a significantly lower value (*p* < 0.05). Gansu *Pennisetum* research revealed that alterations in temperature and nitrogen fertilizer had no significant impact on organic carbon, total phosphorus, and total potassium levels; however, they did exert a significant influence on pH and total nitrogen content. Specifically, the pH of the N1T2 treatment was found to be significantly higher than that of other treatments (*p* < 0.05), while the N3T2 treatment exhibited a notably lower pH value compared to other treatments (*p* < 0.05). Furthermore, it was observed that the total nitrogen content in the N3T2 treatment was considerably higher than that in other treatments; conversely, the N1T3 treatment displayed a markedly lower level of total nitrogen when compared with other treatments (*p* < 0.05).

**Figure 3:**
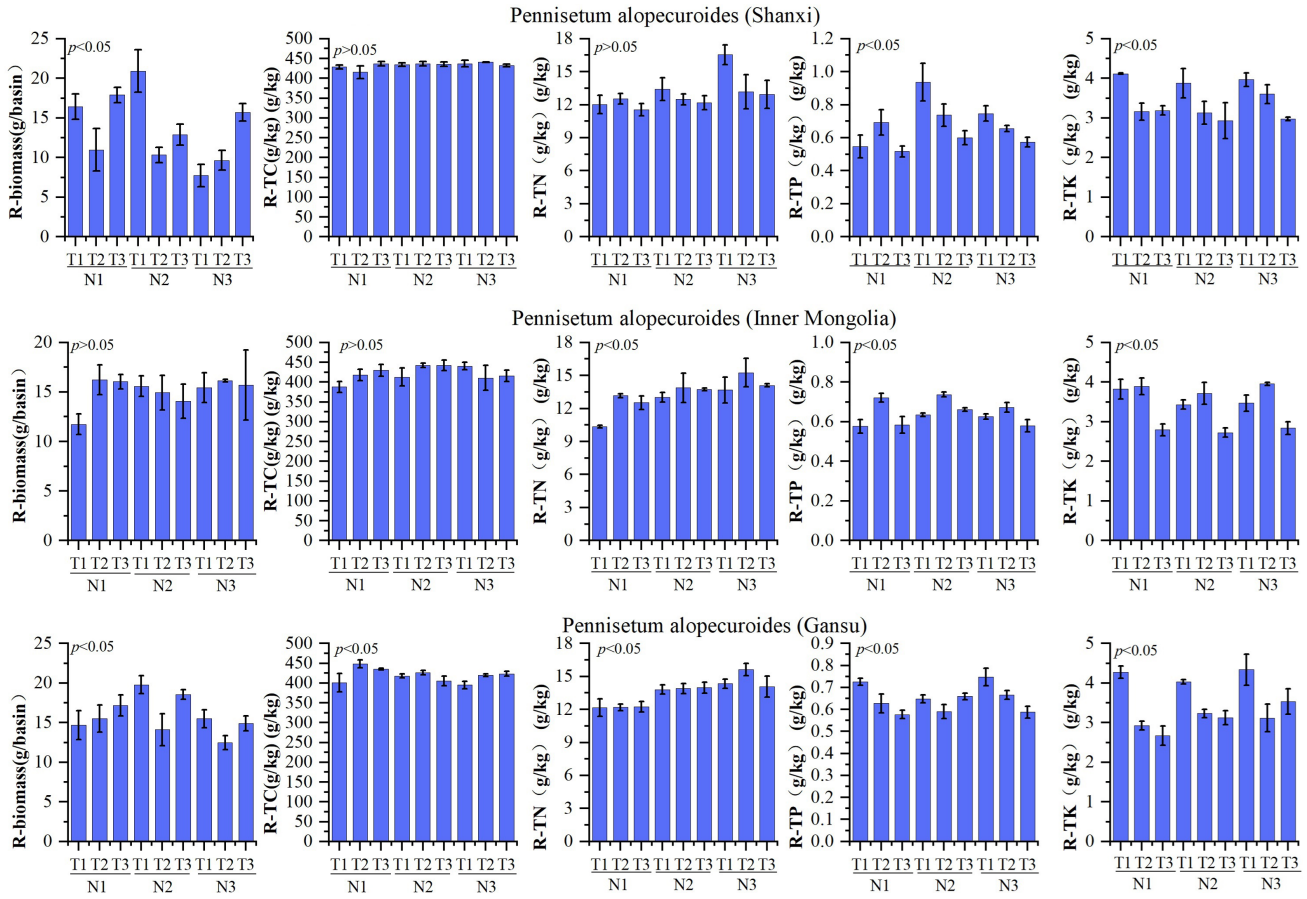
Plant root characteristics of different *Pennisetum alopecuroides* in the trail.

The nutrient composition of *Pennisetum* roots is presented in Figure 3. A study conducted on Shaanxi *Pennisetum* revealed that variations in temperature and nitrogen fertilizer did not have a significant impact on total nitrogen and organic carbon levels, but had a notable influence on biomass, total phosphorus, and total potassium concentrations. The biomass of the N2T1 treatment exhibited significantly higher values compared to other treatments (*p* < 0.05), while the N3T1 treatment showed significantly lower values (*p* < 0.05). Moreover, the N2T1 treatment displayed significantly higher levels of total phosphorus than other treatments, whereas the N1T3 treatment demonstrated significantly lower levels (*p* < 0.05). Additionally, the N1T1 treatment exhibited notably higher amounts of total potassium compared to other treatments, with the N2T3 treatment displaying significantly lower quantities (*p* < 0.05). The study conducted in Inner Mongolia on the Pennisetum species revealed that variations in temperature and nitrogen fertilizer did not exert a significant influence on biomass and organic carbon. However, they had a notable impact on the levels of nitrogen, phosphorus, and potassium. Specifically, the N3T2 treatment exhibited significantly higher total nitrogen content compared to other treatments (*p* < 0.05), while the N1T1 treatment showed significantly lower levels. Similarly, the N2T2 treatment displayed significantly higher total phosphorus content than other treatments (*p* < 0.05), whereas the N3T3 treatment demonstrated significantly lower levels. Moreover, the N1T2 treatment indicated a significant increase relative to other treatments (*p* < 0.05), while the N2T3 treatment exhibited a significant decrease (*p* < 0.05). Gansu *Pennisetum* study found that temperature and nitrogen changes significantly affected biomass, organic carbon, total nitrogen, total phosphorus, and total potassium. N2T1 treatment had significantly higher biomass than other treatments, while N3T2 treatment had significantly lower biomass than other treatments (*p* < 0.05). N1T2 treatment had significantly higher organic carbon than other treatments, while N1T1 treatment had significantly lower organic carbon than other treatments (*p* < 0.05). N3T2 treatment had significantly higher total nitrogen than other treatments, while N1T2 treatment had significantly lower total nitrogen than other treatments (*p* < 0.05). N3T1 treatment had significantly higher total phosphorus than other treatments, while N1T3 treatment had significantly lower total phosphorus than other treatments (*p* < 0.05). N3T1 treatment had significantly higher total potassium than other treatments, while N1T3 treatment had significantly lower total potassium than other treatments (*p* < 0.05).

**Figure 4:**
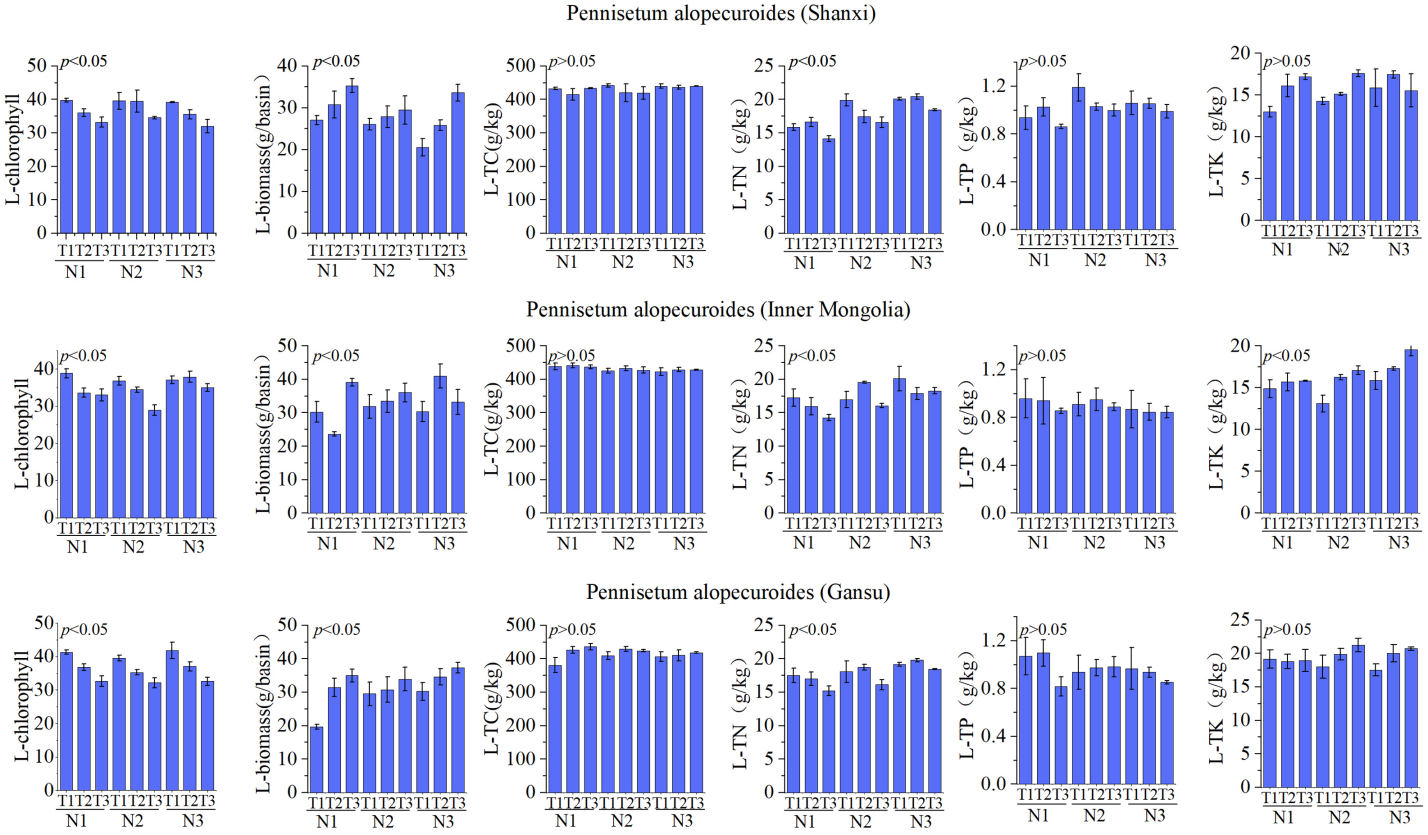
Physical and chemical properties of leaves.

The leaf nutrient factors of *Pennisetum* are illustrated in Figure 4. A study conducted in Shaanxi revealed that variations in temperature and nitrogen fertilizer had no significant impact on organic carbon, total phosphorus, and total potassium levels. However, they did significantly influence chlorophyll content, biomass production, and total nitrogen concentration. Specifically, the N1T1 treatment exhibited significantly higher chlorophyll levels compared to other treatments, the N1T3 treatment resulted in significantly greater biomass production than other treatments, and the N3T2 treatment showed a significantly higher total nitrogen concentration (*p* < 0.05). Similarly, an investigation carried out in Inner Mongolia demonstrated that changes in temperature and nitrogen fertilizer had no notable effect on organic carbon or total phosphorus levels but did have a significant impact on chlorophyll content, biomass production, total nitrogen concentration as well as total potassium levels.The chlorophyll content in the N1T1 treatment exhibited a significant increase compared to other treatments, while the biomass in the N3T2 treatment showed a significantly higher value than other treatments. Moreover, the total nitrogen concentration was found to be significantly elevated in the N3T1 treatment relative to other treatments, and similarly, the total potassium level was notably higher in the N3T3 treatment (*p* < 0.05). The Gansu *Pennisetum* study revealed that variations in temperature and nitrogen fertilizer application did not exert a significant influence on organic carbon, total phosphorus, and total potassium levels. However, these factors had a pronounced impact on chlorophyll content, biomass production, and total nitrogen concentration. Specifically, the chlorophyll content of plants subjected to the N3T1 treatment displayed a significant increase compared to other treatments. Additionally, plants treated with N3T3 exhibited significantly higher biomass values than those under different conditions. Furthermore, an elevated level of total nitrogen was observed in plants treated with N3T2 when compared to other treatments (*p* < 0.05).

### 3.2 Structural composition of soil microbial communities

**Figure 5:**
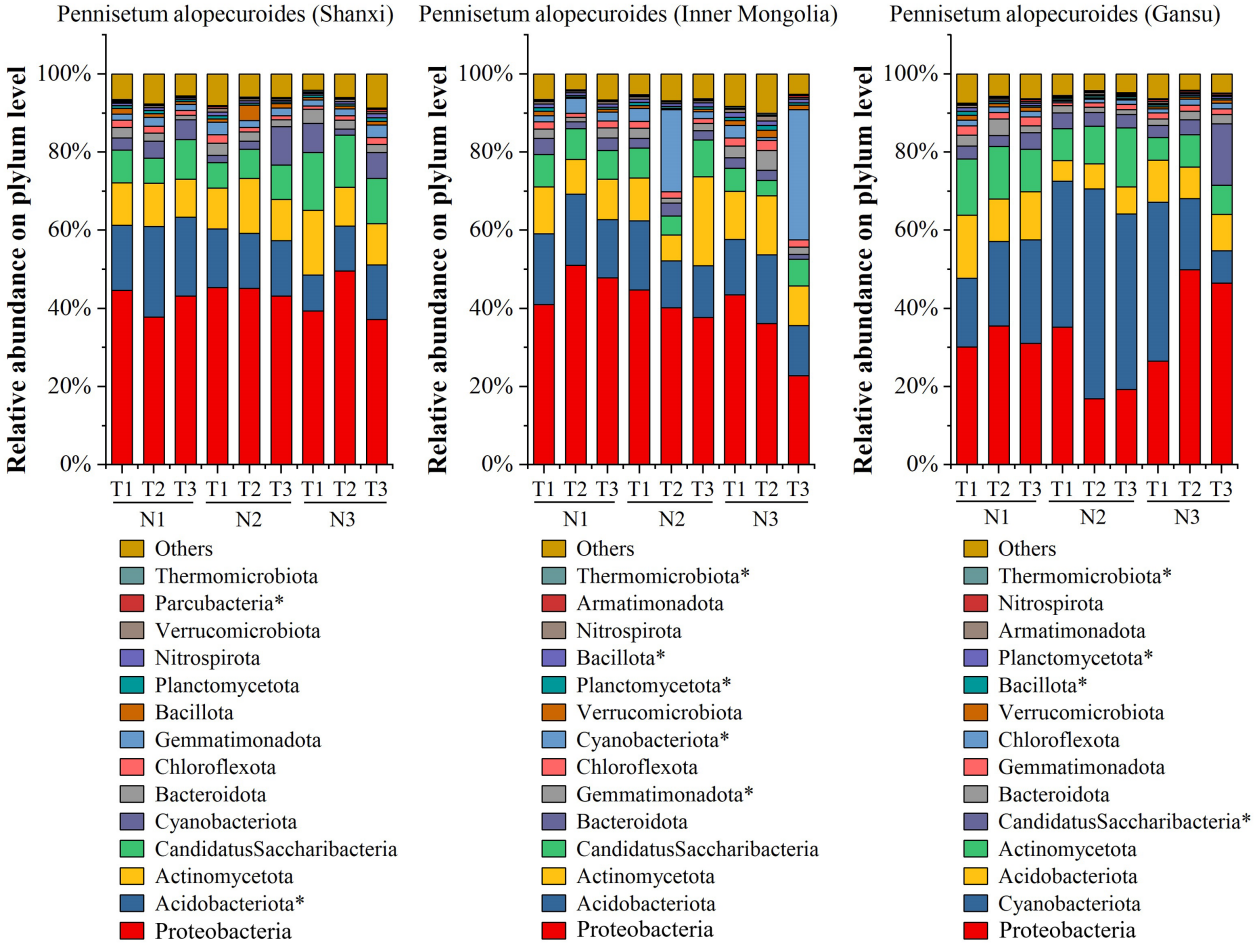
Soil bacterial community composition. * indicates significant differences between treatments (*p* < 0.05).

By employing high-throughput sequencing, we investigated the soil bacterial community of *Pennisetum* from three different seed sources (Figure 5). At the phylum taxonomic level, our results revealed that *Proteobacteria*, *Acidobacteriota*, *Actinomycetota*, *Candidatus Saccharibacteria*, *Cyanobacteriota*, *Bacteroidota* and *Chlorobiota* were the predominant taxa in the *Pennisetum* soil bacterial community under varying temperatures and nitrogen fertilizer levels. Among these phyla, *Proteobacteria* exhibited the highest relative abundance at 49.63% in the N3T2 treatment and lowest at 37.16% in the N3T3 treatment; whereas *Acidobacteriota* showed its highest relative abundance at 23.22% in the N1T2 treatment and lowest at 9.13% in the N3T1 treatment. Furthermore, significant differences were observed in the abundance of *Acidobacteriota* and *Parcubacteria* among treatments (*p* < 0.05). In the study on *Pennisetum* in Inner Mongolia, the soil bacterial community associated with *Pennisetum* under varying temperatures and nitrogen fertilizer levels was found to be primarily composed of *Proteobacteria*, *Acidobacteriota*, *Actinomycetota*, *Candidatus Saccharibacteria*, *Cyanobacteriota*, *Bacteroidota*, *Gemmatimonadota* and *Chlorobiota* taxa. Among these phyla, *Proteobacteria* exhibited the highest relative abundance ranging from 51.09% (N1T2) to 22.84% (N3T3), while *Acidobacteriota* showed the highest relative abundance at 18.14% (N1T2) and lowest at 11.99%. In addition, *Gemmatimonadota*, *Cyanobacteriota*, *Planctomycetota*, *Bacillota* and *Thermomicrobiota* were significantly different among treatments (*p* < 0.05). For Gansu *Pennisetum*, it was found that the soil bacterial community of *Pennisetum* under different temperatures and nitrogen fertilizer levels mainly included *Proteobacteria*, *Cyanobacteriota*, *Acidobacteriota*, *Actinomycetota*, *CandidatusSaccharibacteria*, *Bacteroidota*, *Gemmatimonadota* and *Chlorobiota* taxa. The first three phyla were dominant, with total relative abundance of 63.82% (N1T1), 67.98% (N1T2), 68.89% (N1T3), 77.87% (N2T1), 76.99% (N2T2), 71.09% (N2T3), and 77.90% (N3T1) at different temperatures and nitrogen fertilization levels, respectively, 76.21% (N3T2), 64.05% (N3T3).The relative abundance of *Cyanobacteriota* was highest at 53.74% in the N2T2 treatment and lowest at 8.28% in the N3T3 treatment, whereas the relative abundance of *Proteobacteria* was highest at 49.94% in the N3T2 treatment and lowest at 16.87% in the N2T2 treatment. In addition, *Candidatus Saccharibacteria*, *Planctomycetota*, *Bacillota* and *Thermomicrobiota* were significantly different (*p* < 0.05) among treatments.

**Figure 6:**
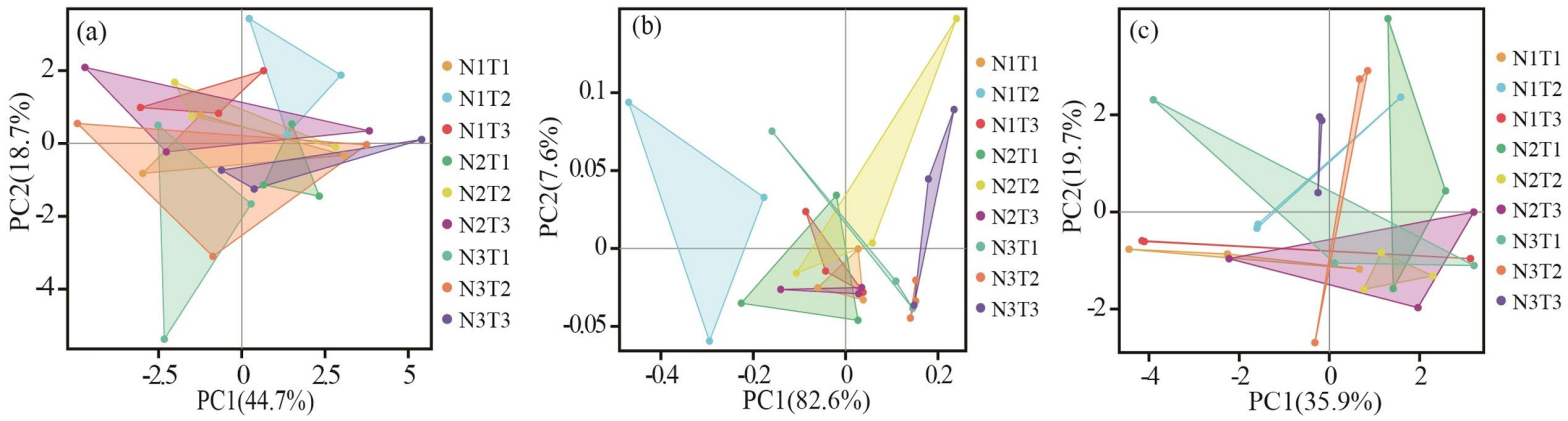
Principal component analysis (PCA) of soil bacteria. Panels **(a–c)** display the PCA for Pennisetum in Shaanxi, Inner Mongolia, and Gansu, respectively.

The results of principal component analysis (Figure 6) revealed distinct variations in soil bacterial communities among three different seed sources of *Pennisetum* under varying temperatures and nitrogen fertilizer levels. Additionally, the ANOSIM test demonstrated significant dissimilarities (*p* < 0.05) in soil microbial communities between *Pennisetum* samples from Inner Mongolia and Gansu at different temperature and nitrogen fertilizer conditions.

**Figure 7:**
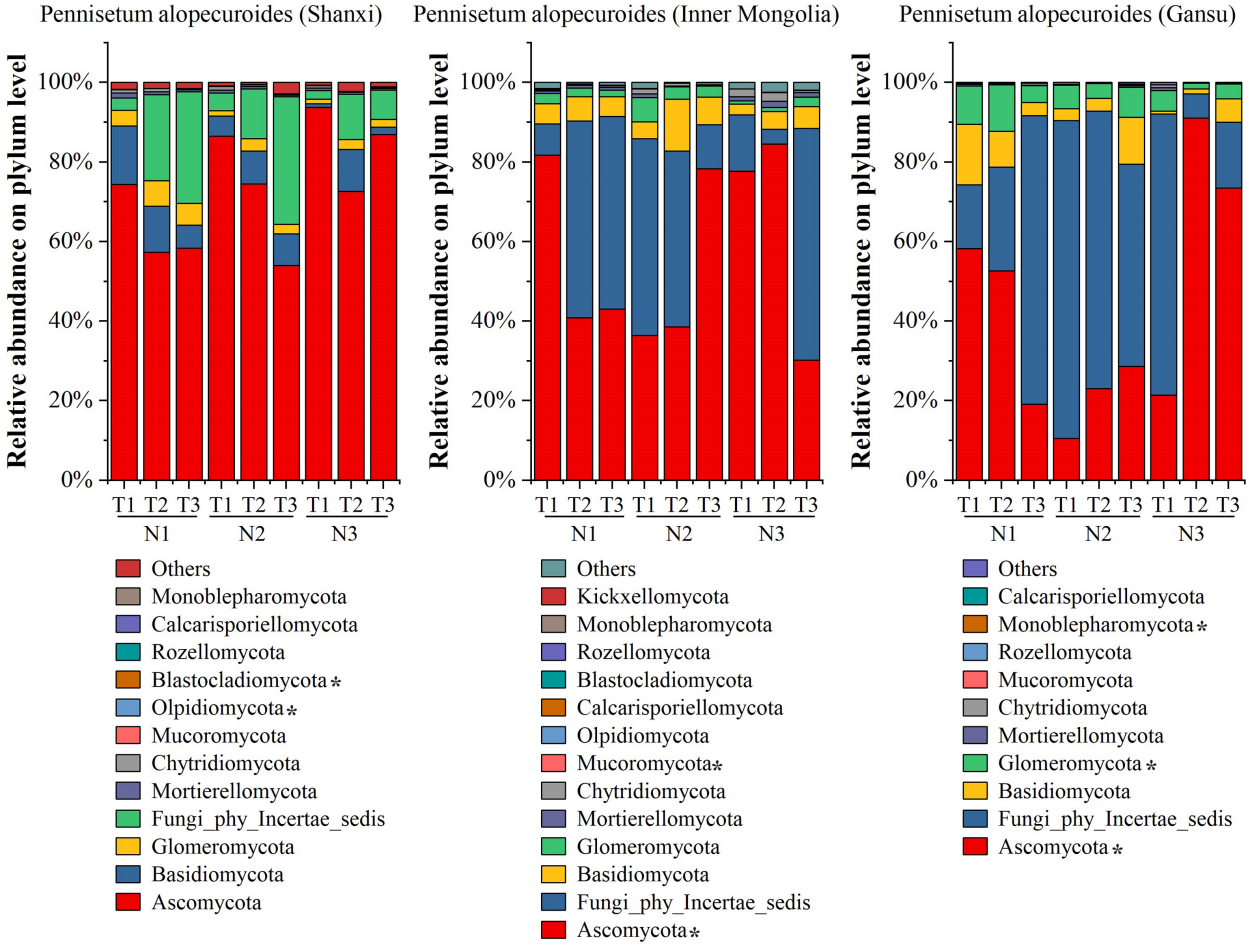
Soil fungal community composition.

High-throughput sequencing of soil fungi from three different provenances of Pennisetum revealed (Figure 7) at the phylum level that Shaanxi *Pennisetum* soil fungal communities included *Ascomycota*, *Basidiomycota*, *Glomeromycota*, *Fungi_phy_Incertae_sedis*, *Mortierellomycota*, *Chytridiomycota*, *Mucoromycota*, and *Olpidiomycota*. *Ascomycota* was the dominant phylum, with relative abundances of 74.39% (N1T1), 57.35% (N1T2), 58.37% (N1T3), 86.51% (N2T1), 74.52% (N2T2), 54.02% (N2T3), 93.76% (N3T1), 72.57% (N3T2), and 86.88% (N3T3). In addition, *Olpidiomycota* and *lastocladiomycota* were significantly different among treatments (*p* < 0.05). For the Inner Mongolian Pennisetum study, *Ascomycota* and *Fungi_phy_Incertae_sedis* were found to be the dominant phylum in each treatment and the abundance was 89.63% (N1T1), 90.07% (N1T2), 91.43% (N1T3), 85.81% (N2T1), and 82.77% (N2T2), respectively, 89.42% (N2T3), 91.84% (N3T1), 88.23% (N3T2), 88.42% (N3T3). The relative abundance of Ascomycota was highest at 84.54% in the N3T2 treatment and lowest at 30.15% in the N3T3 treatment. Additionally, there were significant differences in the abundance of *Ascomycota* and *Mucoromycota* among treatments (*p* < 0.05). For Gansu *Pennisetum*, both *Ascomycota* and Fungi_phy_Incertae_sedis were dominant phyla in each treatment, with abundances of 74.25% (N1T1), 78.73% (N1T2), 91.69% (N1T3), 90.46% (N2T1), and 92.85% (N2T2) respectively, as well as 79.42% (N2T3), 92.06% (N3T1),97.21% (N3T2), and 90.04% (N3T3).The relative abundance of *Ascomycota* was highest at 91.01% in the N3T2 treatment and lowest at 10.53% in the N2T1 treatment. Additionally, there were significant differences in the abundance of *Ascomycota*, *Glomeromycota*, and *Monoblepharomycota* among treatments (*p* < 0.05).

**Figure 8:**
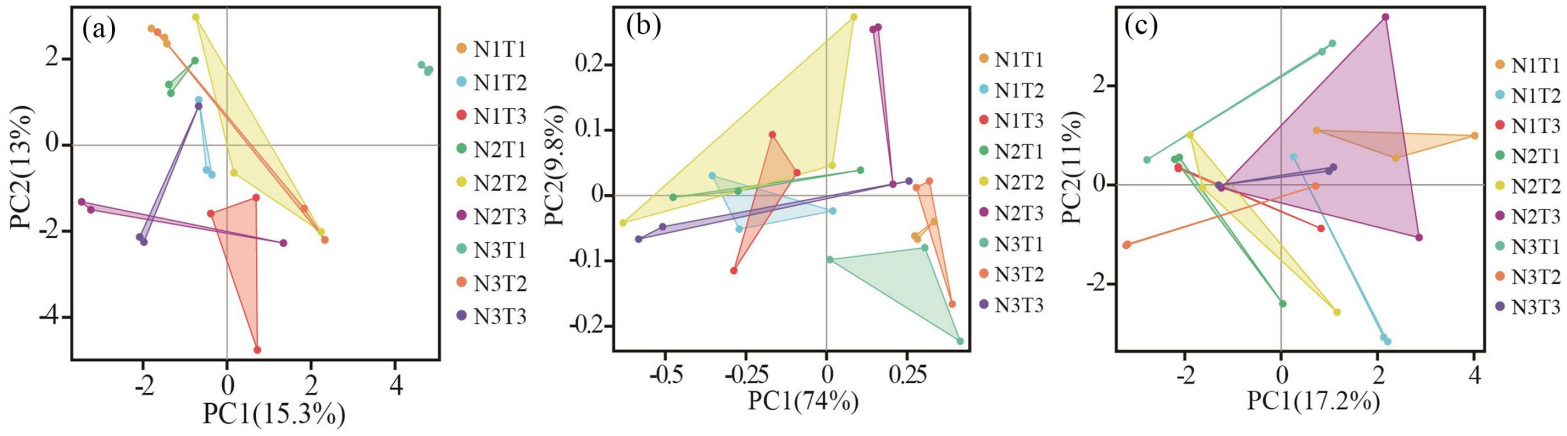
Principal component analysis (PCA) of soil fungi. Panels **(a–c)** display the PCA for Pennisetum in Shaanxi, Inner Mongolia, and Gansu, respectively.

The results of principal component analysis (Figure 8) revealed distinct variations in soil fungal communities among three different seed sources of *Pennisetums* under varying temperatures and nitrogen fertilizer levels. Additionally, the ANOSIM test demonstrated significant differences (*p* < 0.05) in soil microbial communities among the three seed sources of *Pennisetums* at different temperature and nitrogen fertilizer combinations.

### 3.3 Soil microbial diversity

**Figure 9:**
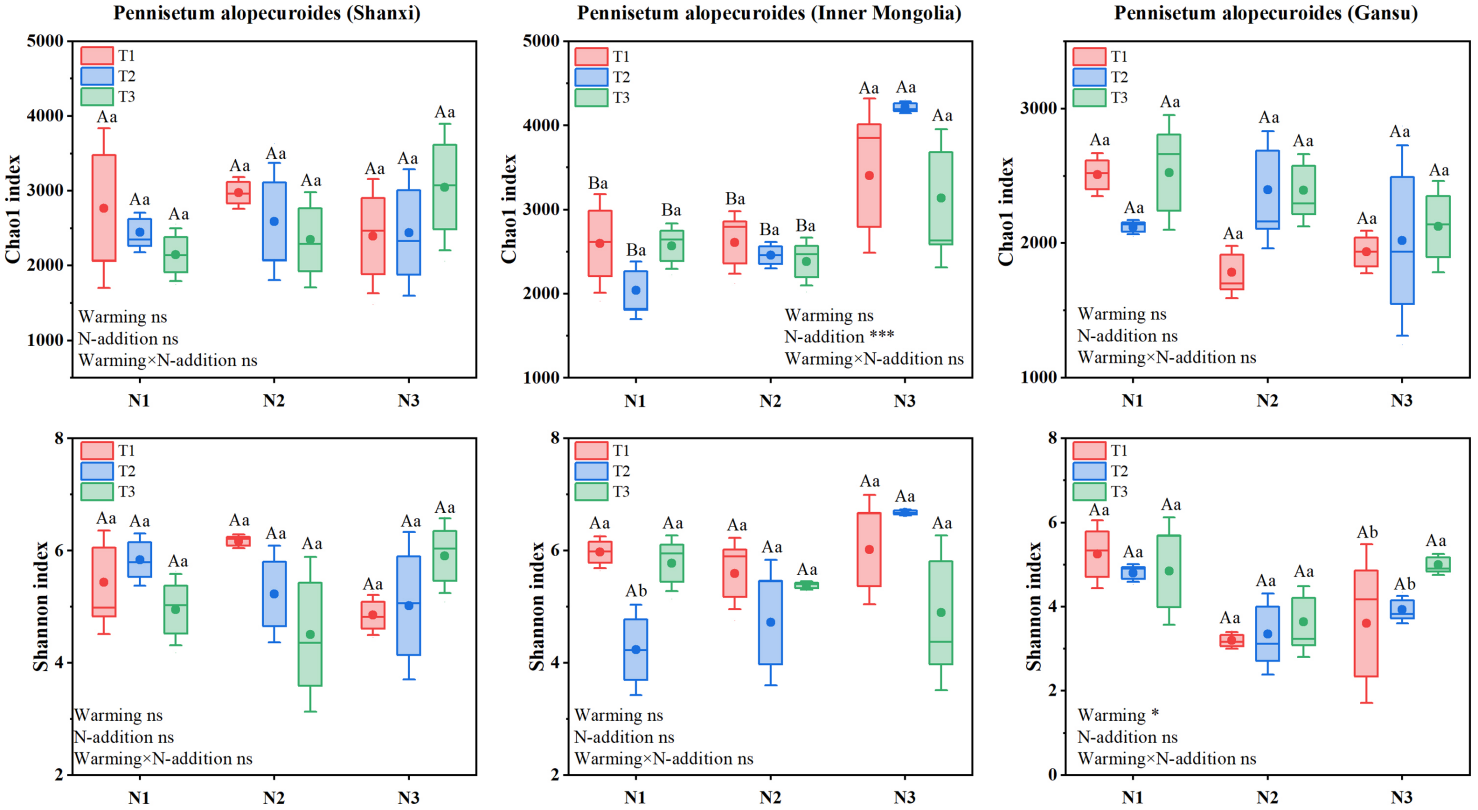
Soil bacterial community diversity index.

Soil bacterial *α*-diversity (Figure 9) showed that Chao1 and Shannon indices for Shaanxi *Pennisetum* soil bacteria were higher in N2T1 treatment than other treatments. Inner Mongolia *Pennisetum* soil bacterial Chao1 and Shannon indices were higher in N3T2 treatment than other treatments and lower in N1T2 treatment. Gansu *Pennisetum* soil bacterial Chao1 and Shannon indices were higher in N1T1 treatment than other treatments and lower in N2T1 treatment.

**Figure 10:**
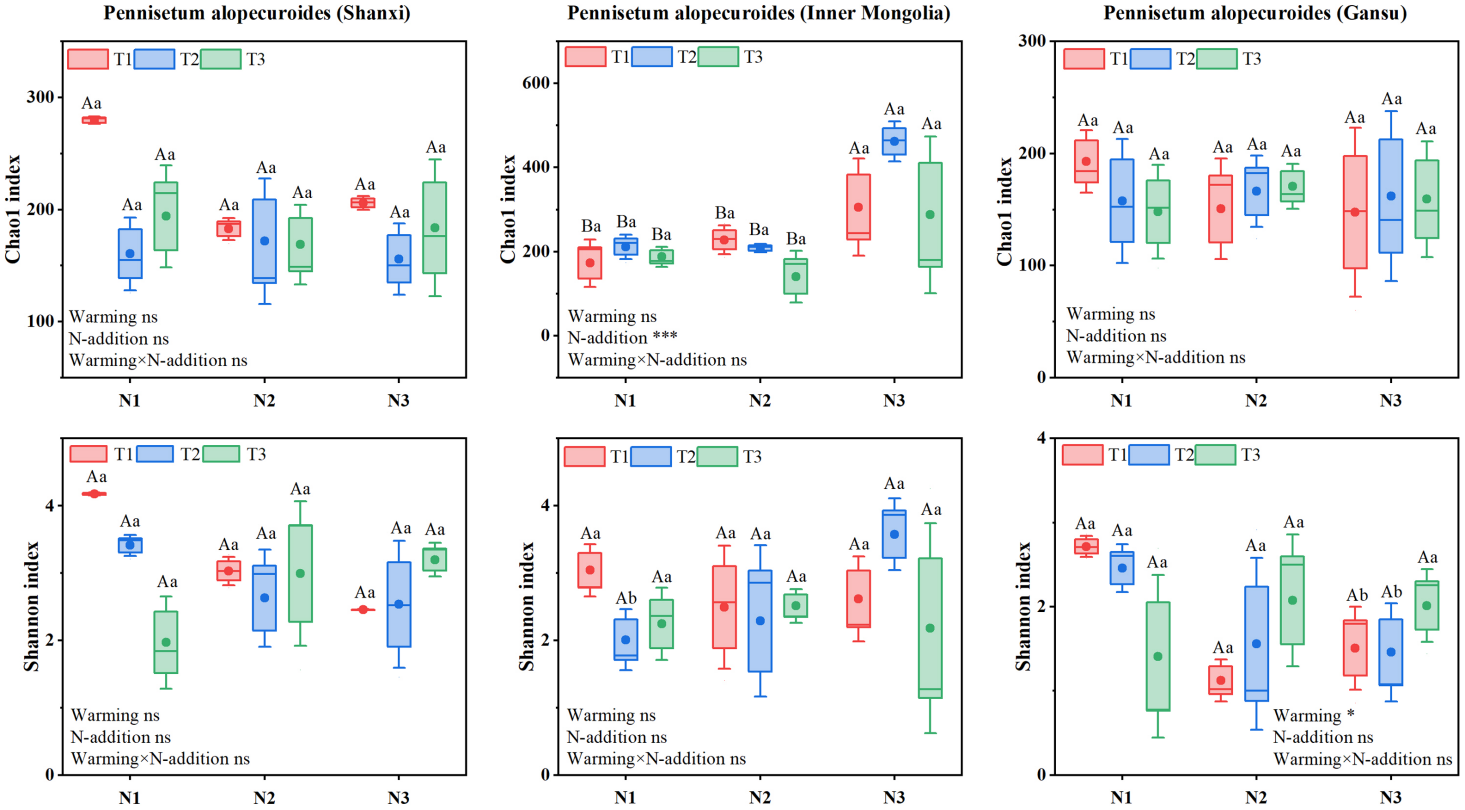
Soil fungal community diversity index.

Soil fungal α-diversity (Figure 10) showed that Chao1 index for Shaanxi *Pennisetum* soil fungi was higher in N3T1 treatment than other treatments, while Shannon index was higher in N3T3 treatment. Inner Mongolia *Pennisetum* soil fungal Chao1 and Shannon indices were higher in N3T2 treatment than other treatments. Gansu *Pennisetum* soil fungal Chao1 and Shannon indices were higher in N1T1 treatment than other treatments and lower in N2T1 treatment (see Figure 10).

### 3.4 Soil microbial community co-occurrence patterns

**Figure 11:**
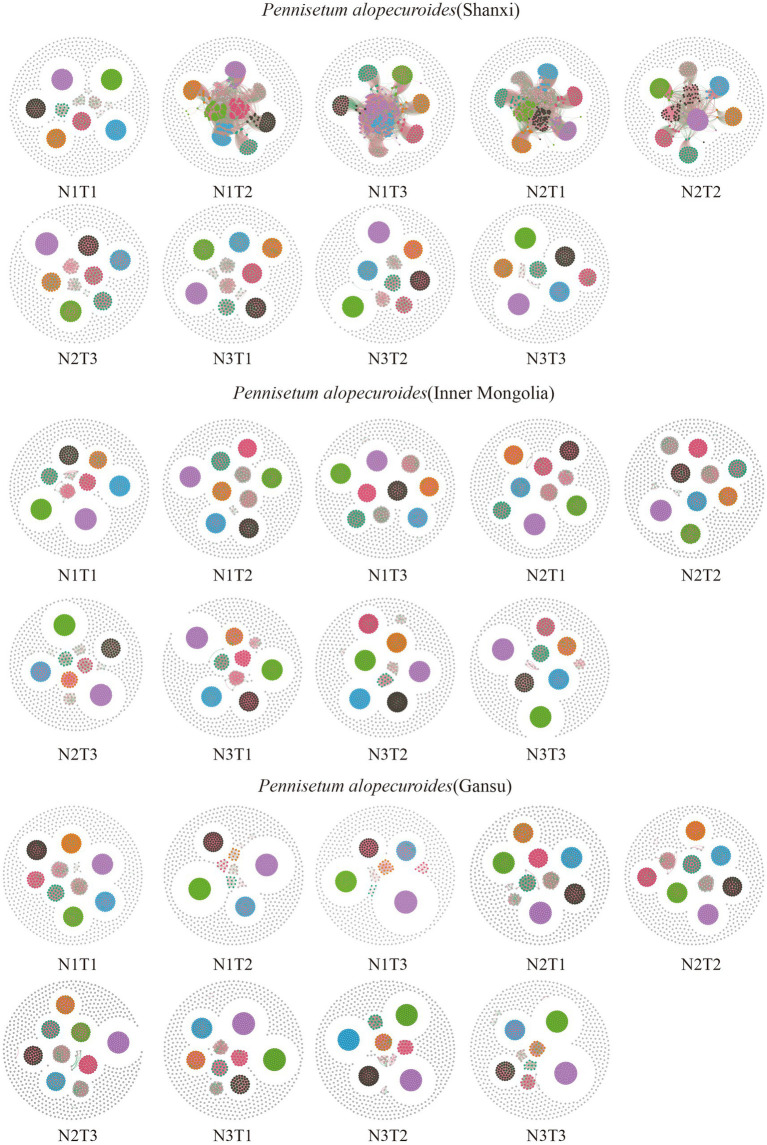
Co-linear network analysis of soil microbial communities. The node size represents the degree (or the number of nodes connected to it). Red connection lines are positive connections, green connection lines are negative connections. The number of nodes is colored according to the different classifications.

The microbial co-occurrence network characteristics of *Pennisetum* soil from three different seed sources exhibited significant differences (Figure 11). Analysis of the microbial network characteristics in Shaanxi *Pennisetum* soil revealed that the N3T3 treatment displayed a higher number of edges (18955), nodes (995), average weighted degree, and graph density in the microbial co-occurrence network (Table 2). These findings indicate that the soil microbial co-occurrence network in this treatment is more complex with intricate interactions among different species, while also exhibiting a higher degree of modularity and distinct sub-structures. Moreover, the N1T1 treatment exhibited a lower proportion of positive correlation edges and a higher proportion of negative correlation edges compared to other treatments. Conversely, the N3T2 treatment displayed a higher proportion of positive correlation edges and a lower proportion of negative correlation edges in comparison to other treatments. These findings suggest that there exists a robust collaborative relationship among species within microbial communities subjected to high-concentration nitrogen additions and high-temperature treatments, while a strong competitive relationship is observed among species within microbial communities exposed to low-concentration nitrogen additions and low-temperature treatments. The relationship between microbial communities in the low-temperature and high-temperature treatments exhibited a robust association. Investigation and analysis of soil microbial networks in Inner Mongolia *Pennisetum* (Table 3) revealed that the N3T3 treatment displayed higher numbers of edges (19875), nodes (998), average weighted degree, and graph density within the microbial co-occurrence network, indicating increased complexity of species interactions. Conversely, the N1T2 treatment demonstrated higher modularity with evident substructure. Moreover, the N3T2 treatment exhibited a lower proportion of positive correlation edges and a higher proportion of negative correlation edges compared to the other treatments. Conversely, the N3T3 treatment showed a higher proportion of positive correlation edges and a lower proportion of negative correlation edges compared to the other treatments. These findings suggest that there exists a strong collaborative relationship among species within microbial communities under high nitrogen concentration and high temperature conditions, while a strong competitive relationship is observed between species in microbial communities subjected to high nitrogen concentration and low temperature conditions. The association between microbial communities under high nitrogen concentration and low temperature conditions was found to be significant. The research and analysis of soil microbial network characteristics in Gansu *Pennisetum* (Table 4) revealed that the N1T3 treatment exhibited a higher number of edges (30222), nodes (980), average weighted degree, and graph density in the microbial co-occurrence network. This indicates that the soil microbial co-occurrence network in this treatment displayed greater complexity and more intricate interactions among different species. Conversely, the N2T2 treatment demonstrated a higher degree of modularity with evident substructures. Furthermore, compared to other treatments, the N3T3 treatment had a lower proportion of positive correlation edges and a higher proportion of negative correlation edges, while the N3T1 treatment showed a higher proportion of positive correlation edges and a lower proportion of negative correlation edges. These findings suggest that there exists strong collaboration between species within high-concentration nitrogen additions and low-temperature treatments, whereas competitive relationships prevail among species within high-concentration nitrogen additions and high-temperature treatments. Notably, the negative correlation margins were lower than those observed in other treatments.

**Table 2:** Topological parameters of microbial co-occurrence network in Shaanxi *Pennisetum* soil.

Topological parameters	N1T1	N1T2	N1T3	N2T1	N2T2	N2T3	N3T1	N3T2	N3T3
Node	992	996	957	998	998	916	938	982	995
Edge	17,033	16,571	16,017	16,356	16,595	14,633	13,138	17,600	18,955
Positive edge	52.17%	66.92%	70.04%	63.84%	75.14%	69.7%	58.32%	86.45%	70.82%
Negative edge	47.83%	33.08%	29.96%	36.16%	24.86%	30.3%	41.68%	13.55%	29.18%
Average weighting	17.17	17.101	17.798	17.067	17.067	15.975	14.006	17.923	19.05
Graph density	0.017	0.017	0.018	0.016	0.017	0.017	0.015	0.018	0.019
Modularity	0.800	0.742	0.646	0.713	0.755	0.763	0.829	0.764	0.77

**Table 3:** Topological parameters of the microbial co-occurrence network of *Pennisetum* soil in Inner Mongolia.

Topological parameters	N1T1	N1T2	N1T3	N2T1	N2T2	N2T3	N3T1	N3T2	N3T3
Node	996	992	996	998	994	908	998	1,000	998
Edge	18,828	13,891	14,257	15,818	13,945	16,480	19,189	17,325	19,875
Positive edge	69.11%	68.00%	67.03%	62.83%	75.15%	57.49%	77.66%	54.81%	78.03%
Negative edge	30.89%	32.00%	32.97%	37.17%	24.85%	42.51%	22.34%	45.19%	21.97%
Average weighting	18.904	14.003	14.314	15.85	14.029	18.15	19.227	17.325	19.915
Graph density	0.019	0.014	0.014	0.016	0.014	0.02	0.019	0.017	0.02
Modularity	0.756	0.855	0.85	0.796	0.841	0.719	0.740	0.827	0.755

**Table 4:** Topological parameters of microbial co-occurrence network of *Pennisetum* soil in Gansu.

Topological parameters	N1T1	N1T2	N1T3	N2T1	N2T2	N2T3	N3T1	N3T2	N3T3
Node	998	988	980	926	1,000	998	944	986	974
Edge	15,033	27,004	30,222	13,776	14,334	15,629	17,470	21,136	24,318
Positive edge	69.07%	65.61%	64.04%	70.2%	79.98%	78.26%	87.49%	67.37%	58.48%
Negative edge	30.93%	34.39%	35.96%	29.8%	20.02%	21.74%	12.51%	32.63%	41.52%
Average weighting	15.063	27.332	30.839	14.877	14.334	15.66	18.506	21.436	24.967
Graph density	0.015	0.028	0.032	0.016	0.014	0.016	0.02	0.022	0.026
Modularity	0.839	0.65	0.516	0.819	0.855	0.762	0.728	0.75	0.633

### 3.5 Relationship between soil microbial communities and physicochemical factors

**Figure 12:**
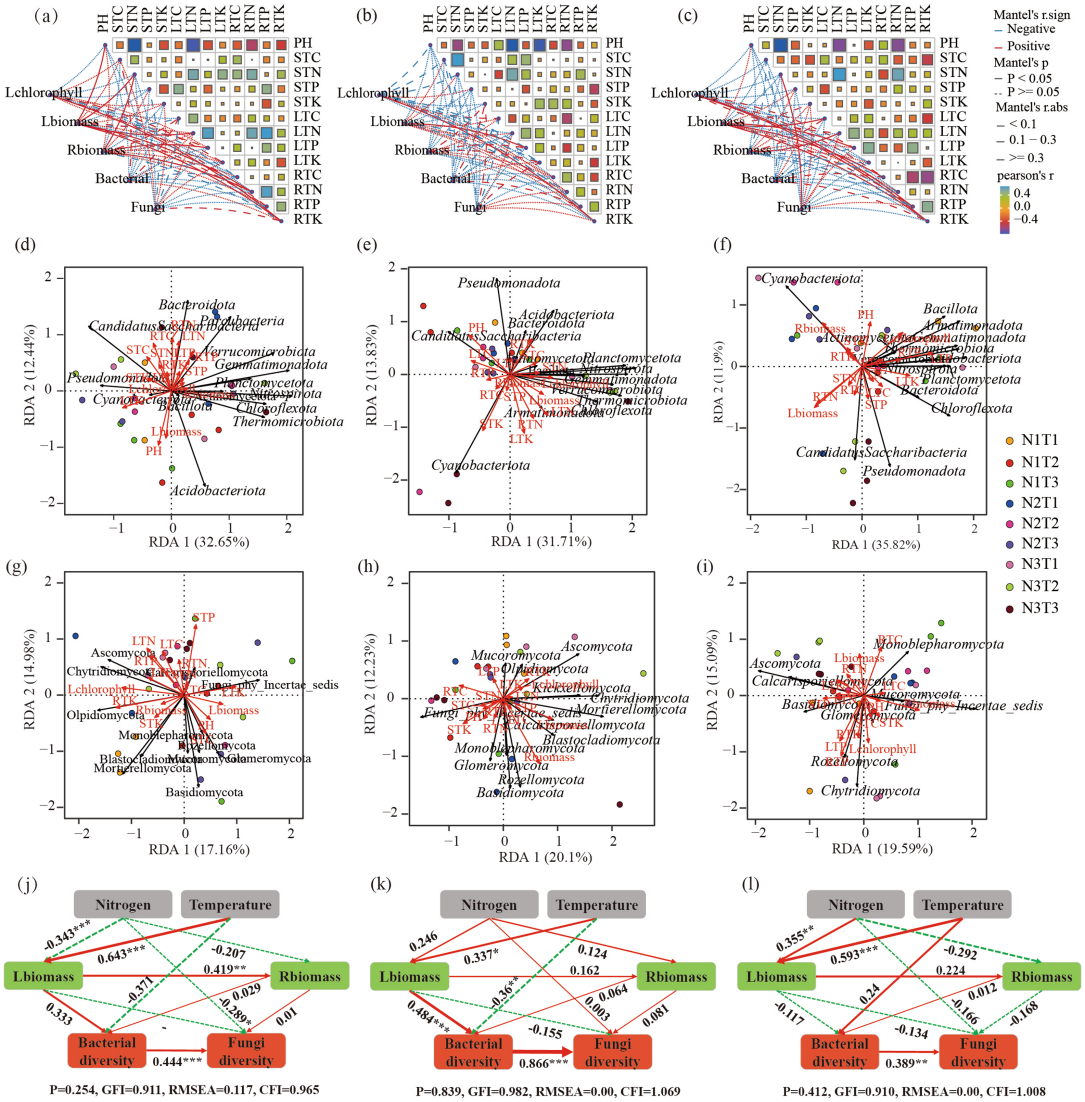
Panels **(a–c)** display the Mantel analysis for Pennisetum in Shaanxi, Inner Mongolia, and Gansu, respectively. Panels **(d–f)** show the redundancy analysis (RDA) of bacterial communities and physicochemical factors for Pennisetum in Shaanxi, Inner Mongolia, and Gansu, respectively. Panels **(g–i)** illustrate the RDA of fungal communities and physicochemical factors in Shaanxi, Inner Mongolia, and Gansu, respectively. Panels **(j–l)** depict the structural equation models for Pennisetum in Shaanxi, Inner Mongolia, and Gansu, respectively.

The Mantel analysis (Figures 12a–c) revealed a significant positive correlation (*p* < 0.05) between Shaanxi *Pennisetum* L-chlorophyll and R-TK. Additionally, there was a significant positive correlation (*p* < 0.05) between L-biomass and soil pH, S-TN, L-TN, R-TN, as well as R-TK. Moreover, R-biomass showed a significant positive correlation (*p* < 0.05) with S-TN, while both L-TP and R-TN exhibited a significant positive correlation (*p* < 0.05). In the case of Inner Mongolia *Pennisetum*, there was a significant positive correlation (*p* < 0.05) between L-chlorophyll and R-TK; similarly, R-biomass displayed a significant positive correlation with soil L-TP (*p* < 0.05). Furthermore, Gansu *Pennisetum* demonstrated a significant positive correlation (*p* < 0.05) between L-chlorophyll and both R-TP and R-TK; additionally, there was a significant positive association (*p* < 0.05) between L-biomass and soil variables such as L-TC, L-TP, R-TK, and R-TC. Finally, the fungal community diversity exhibited a significantly positive correlation (*p* < 0.05) with S-TN.

Structural equation modeling analysis (Figures 12j–l) found that L-biomass and R-biomass were significantly positively correlated in Shaanxi *Pennisetum*, and nitrogen addition significantly affected fungal diversity (*p* < 0.05). In Inner Mongolia *Pennisetum*, L-biomass and temperature significantly affected bacterial diversity (*p* < 0.05). In Gansu *Pennisetum*, temperature and nitrogen had no significant effect on microorganisms, indicating that soil microbial communities were influenced by multiple factors.

Further redundancy analysis (Figures 12d–i) found that soil pH was the main environmental factor affecting bacteria in Shaanxi *Pennisetum*, and soil total phosphorus was the main environmental factor affecting fungi. In Inner Mongolia *Pennisetum*, soil pH and soil total potassium were the main environmental factors affecting bacteria, and R-biomass was the main environmental factor affecting fungi. In Gansu *Pennisetum*, R-biomass, L-biomass, and L-TP were the main environmental factors affecting bacteria, and R-TC and R-TP were the main environmental factors affecting fungi.

## 4 Discussion

### 4.1 Effects of different nitrogen fertilizers and temperatures on the characteristics of microbial communities in *Pennisetum* soils

Soil microorganisms exhibit diminutive morphology, wide distribution, relatively short life cycles, remarkable diversity, prompt response to environmental perturbations, and the ability to swiftly adapt to environmental changes while upholding ecosystem stability (Xiong et al., 2017). This study showed that *Proteobacteria* in the soil of Shaanxi and Inner Mongolia *Pennisetum* significantly decreased under high temperature and high nitrogen treatments, while *Acidobacteriota* had higher relative abundance under low nitrogen treatment. This may be due to the poor adaptability of *Proteobacteria* to high temperature and high nitrogen environments, inhibiting their metabolic activities and affecting their physiological activities. In contrast, *Acidobacteriota* has a strong adaptability to low nitrogen environments, possibly being more competitive in resource-scarce environments (Li et al., 2022). Additionally, *Cyanobacteriota* in Inner Mongolia *Pennisetum* soil was significantly higher under high temperature and high nitrogen treatments, possibly due to the nitrogen-fixing ability of *Cyanobacteriota*, which helps improve soil nitrogen utilization efficiency under high nitrogen treatments, benefiting their growth and reproduction (Cole et al., 2016). *Cyanobacteriota* can survive and reproduce at higher temperatures. High temperatures may promote the growth and metabolic activities of *Cyanobacteriota*, increasing their relative abundance in high temperature environments (Tang et al., 2002). In Gansu *Pennisetum* soil, *Proteobacteria* had the lowest relative abundance under medium temperature and medium nitrogen treatments, while *Cyanobacteriota* had the highest relative abundance, possibly due to the higher altitude of Gansu *Pennisetum*, causing different adaptability to environmental conditions, resource utilization efficiency, and competition with other microorganisms. Additionally, *Ascomycota* was significantly higher under low temperature and high nitrogen treatments, while *Basidiomycota* and *Glomeromycota* were significantly higher under high temperature and low nitrogen treatments in the soil of the three provenances of *Pennisetum*. This may be due to high nitrogen treatments promoting the secretion of more amino acids and sugars by plant roots, increasing soil nutrient content and further enhancing the reproduction and growth of *Ascomycota*. Low temperature environments cause fungi to produce cold-tolerant enzymes and antifreeze proteins, enabling them to adapt to lower temperature environments and occupy ecological advantages. Low temperature environments may inhibit the growth of some other fungi, allowing *Ascomycota* to dominate in competition (Chen et al., 2017; Hu et al., 2014; Tedersoo et al., 2014). Many species of *Basidiomycota* can grow and reproduce in high temperature environments. These fungi can tolerate higher temperature conditions through heat shock proteins and other protective mechanisms, occupying ecological advantages in high temperature environments. Similarly, some fungi in *Glomeromycota* can maintain activity and reproductive capacity under high temperature conditions (Huang et al., 2023; Vedenicheva, et al., 2018). Low nitrogen treatments reduce the available nitrogen content in soil, benefiting fungi that can efficiently utilize limited nitrogen sources. Many species in *Basidiomycota* and *Glomeromycota* can obtain the necessary nitrogen from organic matter through efficient nitrogen metabolism pathways, gaining advantages under low nitrogen conditions (Hestrin et al., 2021; Pellitier and Zak, 2018). Additionally, high temperature and low nitrogen treatments may change the physical and chemical properties of soil, such as pH, moisture content, and organic matter decomposition rate. These changes affect the ecological niches and competition relationships of different fungal groups. *Basidiomycota* and *Glomeromycota* may have strong adaptability under these changing conditions, gaining competitive advantages in high temperature and low nitrogen environments (Classen et al., 2016; Harrower and Gilbert, 2021).

Redundancy analysis found that soil pH was the main factor affecting soil bacterial communities in Shaanxi and Inner Mongolia *Pennisetum*. Changes in soil acidity and alkalinity can broadly affect bacterial survival and metabolic activities, altering the physiological activities, nutrient forms, and availability of soil microorganisms, indirectly affecting their growth (Ullah et al., 2020). Redundancy analysis found that soil pH was the main factor affecting soil bacterial communities in Shaanxi and Inner Mongolia *Pennisetum*. Changes in soil acidity and alkalinity can broadly affect bacterial survival and metabolic activities, altering the physiological activities, nutrient forms, and availability of soil microorganisms, indirectly affecting their growth (Clayton et al., 2021) Root biomass and aboveground biomass also significantly impact soil microbial communities. Plants provide abundant carbon sources and nutrients for soil microorganisms through root exudates and litter, affecting microbial growth and metabolic activities. Changes in root and aboveground phosphorus content also indirectly affect microbial community structure by altering the distribution and availability of phosphorus in soil (Wang et al., 2023). In summary, the impact of soil environmental factors on microbial communities under different treatments showed significant differences. These differences reflect the characteristics of different treatment soils and reveal the sensitivity and adaptability of microbial communities to environmental changes. Understanding these environmental factors’ impact on microbial communities is crucial for soil management and agricultural production. Optimizing soil pH, rationally applying nutrients, and improving plant growth conditions can effectively regulate soil microbial communities, enhancing soil health and agricultural productivity. The Chao1 and Shannon indices for Shaanxi *Pennisetum* soil bacteria in N2T1 treatment were higher than other treatments, possibly because moderate nitrogen and low temperature conditions may enhance nutrient cycling and organic matter decomposition, promoting bacterial species richness and diversity (Hu et al., 2022). Inner Mongolia *Pennisetum* soil bacterial and fungal Chao1 and Shannon indices were higher in N3T2 treatment than other treatments, possibly due to high nitrogen and moderate temperature providing sufficient nutrients and suitable environments for soil bacteria and fungi, promoting species richness and diversity (Conant et al., 2011). The temperature of the soil in the Gansu area is the most suitable environment for the soil bacteria and fungi. The Chao1 and Shannon indices of soil bacteria and fungi in Pennisetum in Gansu were lower in the N2T1 treatment than in the other treatments, which may be due to the increase of competitive pressure or the unmet specific needs of some bacteria and fungi, resulting in the decrease of the abundance and diversity of the bacterial and fungal community (Tang et al., 2020) The above results indicated that there were significant differences in soil bacterial and fungal diversity in response to N fertilizer application and temperature conditions in different regions. This may be related to the soil types, climatic conditions and growth characteristics of Pennisetum in each region.

### 4.2 Effects of different nitrogen fertilizers and temperatures on microbial interactions in Pennisetum soils

Molecular ecological networks can reflect interactions between different groups within a community and assess the complexity of target communities, successfully applied to analyze environmental characteristics’ impact on microbial communities (Carr et al., 2019). Soil microbial co-occurrence networks under different nitrogen and temperature levels showed significant differences. This study showed strong cooperative relationships among microbial species under high nitrogen environments in the soil of three provenances of *Pennisetum*, possibly due to high nitrogen environments promoting ecological niche differentiation among microbial species and the production and transmission of signaling molecules and metabolites in microbial communities, further promoting microbial communication and cooperation (Bernard et al., 2022; Lemaire et al., 2021) Shaanxi and Inner Mongolia *Pennisetum* had strong cooperative relationships among soil microbial species under high temperature conditions, while Gansu *Pennisetum* had strong cooperative relationships under low temperature conditions, possibly due to the adaptation of Shaanxi and Inner Mongolia *Pennisetum* soil microorganisms to high temperature environments and Gansu *Pennisetum* soil microorganisms to low temperature environments (Bardgett and Putten, 2014). The *Pennisetum* may secrete diverse chemicals at varying temperatures, and these inter-root secretions can exert influence on the composition and function of microbial communities; inter-root secretions under high temperatures may facilitate the growth of specific collaborative microorganisms, while those under low temperatures may promote the cooperation among another group of microorganisms (Chaparro et al., 2013). In general, future investigations could delve into the molecular mechanisms underlying alterations in microbial network characteristics across different environmental conditions and elucidate their impacts on *Pennisetum* growth and ecosystem functioning. This will help better understand the role of soil microorganisms in agriculture and ecological restoration and provide scientific evidence for optimizing planting and management strategies.

### 4.3 Environmental impacts and limitations of this study

This study explored the characteristics of soil microbial communities, and also used microbial co-occurrence network and pathway analysis to explore the effects of nitrogen fertilizer additions and temperature changes on the stability of the soil ecosystems of *Pennisetum*, among others. It found strong cooperative relationships among microbial species under high nitrogen and high temperature treatments for Shaanxi and Inner Mongolia *Pennisetum*, while Gansu *Pennisetum* showed strong competitive relationships. Therefore, it is necessary to determine the effective improvement of soil ecosystem stability under different nitrogen and temperature treatments for different provenances of *Pennisetum*, thereby improving soil moisture retention and nutrient cycling efficiency (Agnihotri et al., 2022). In recent years, no studies have evaluated the ecological stability of soil microorganisms in different provenances of *Pennisetum*. Ecological network structure analysis provides insights into microbial community interactions and system stability (Zeng et al., 2016). Increased connectivity and complexity of microbial networks under high temperature and high nitrogen treatments indicate increased ecosystem stability and enhanced resistance to ecological disturbances (Wang et al., 2019). Therefore, soil microbes play an important role in the ecosystem.

This study unveiled significant impacts of nitrogen fertilizer and temperature fluctuations on the microbial community structure in *Pennisetum* soils located in Northwest China, indicating that moderate levels of nitrogen fertilizer and temperature can enhance arbuscular mycorrhizal fungi (AMF) diversity and ecosystem functionality. However, improper nitrogen fertilizer application and temperature changes may exert detrimental effects on microbial communities and overall ecosystem health. Therefore, future investigations should prioritize exploring the composition and function of soil microfood webs comprising fungi, bacteria, nematodes, and protozoa to comprehensively comprehend the consequences of nitrogen fertilization and temperature variations on soil microfood webs as well as ecosystem functioning.

## 5 Conclusion

This study deepened our understanding of the mechanisms of soil microbial changes under different nitrogen and temperature conditions. The dominant bacterial and fungal phyla in the soil of three different provenances of *Pennisetum* were *Proteobacteria* and *Ascomycota*, respectively. Nitrogen and temperature variations significantly affected the richness and diversity of soil microbial communities, leading to significant changes in their structure. Ecological network analysis indicated strong cooperative relationships among microbial species under high nitrogen and temperature treatments for Shaanxi and Inner Mongolia *Pennisetum*, while Gansu *Pennisetum* showed strong competitive relationships. Soil pH, total potassium, and total phosphorus were the main environmental factors affecting microorganisms. This experiment explored the microbial community changes and driving factors under different nitrogen fertilizer and temperature levels, which can provide a theoretical basis for evaluating the sustainable use of *Pennisetum* artificial grassland in Northwest China.

## Data Availability

The bacterial and fungal DNA sequences of the 81 soil samples were deposited in the SRA of the NCBI database under accession nos. PRJNA1137271, PRJNA1137536.
